# Fetal demise and Wernicke–Korsakoff syndrome in a patient with hyperemesis gravidarum: a case report

**DOI:** 10.1186/s13256-022-03748-2

**Published:** 2023-02-02

**Authors:** Alisa Olmsted, Andrea DeSimone, Jahaira Lopez-Pastrana, Madeleine Becker

**Affiliations:** 1 Department of Psychiatry and Human Behavior, Thomas Jefferson University Hospital, Philadelphia, PA USA; 2 Department of Psychiatry, Bayhealth Medical Center, Dover, DE USA; 3 Department of Integrative Medicine and Nutritional Sciences, Thomas Jefferson University Hospital, Philadelphia, USA; 4 Present Address: Stanford University and the Sierra-Pacific Mental Illness Research, Education, and Clinical Center (MIRECC), VA Palo Alto Health Care System, Palo Alto, USA

**Keywords:** Wernicke–Korsakoff syndrome, Thiamine, Abortion, Hyperemesis, Case report

## Abstract

**Background:**

Wernicke–Korsakoff syndrome is a neuropsychiatric disorder caused by thiamine deficiency composed of two related disorders accounting for an acute presentation and chronic progression. Hyperemesis gravidarum presents a significant risk factor for Wernicke–Korsakoff syndrome as symptoms may rapidly progress in the setting of pregnancy. We present the first-reported case of hyperemesis-gravidarum-associated Wernicke encephalopathy in a patient in the first half of pregnancy in which a missed diagnosis led to septic shock, fetal demise, and eventual profound Korsakoff syndrome.

**Case presentation:**

We present the case of a 33-year-old primigravid African American woman at 15 weeks gestational age who initially presented at a community emergency department with nausea and vomiting that ultimately progressed to severe hyperemesis-gravidarum-associated Wernicke–Korsakoff syndrome, fetal demise, and septic shock. The patient received a total of 6 weeks of high-dose parenteral thiamine. Magnetic resonance imaging of the head and formal neuropsychological assessment following treatment plateau confirmed the diagnosis of Wernicke–Korsakoff syndrome.

**Conclusions:**

The multisystem complications seen in severe thiamine deficiency can delay timely administration of high-dose thiamine, particularly in pregnancy, in which the classic triad of Wernicke–Korsakoff syndrome may not raise clinical suspicion due to rapid progression of neurological sequelae in this population. We advise a low threshold for parenteral thiamine repletion in pregnant women with persistent vomiting as hyperemesis gravidarum-induced severe thiamine deficiency can result in Wernicke–Korsakoff syndrome, sepsis, and fetal demise.

## Introduction

Wernicke–Korsakoff syndrome (WKS) is a neuropsychiatric disorder composed of two related disorders caused by thiamine deficiency. Wernicke encephalopathy (WE) is characterized by the classic triad of symptoms with acuteonset encephalopathy, ophthalmoplegia, and ataxia. WE may progress to Korsakoff syndrome (KS), which is characterized by marked irreversible deficits in anterograde and retrograde memory, as well as apathy and confabulation. Around 85% of patients with WE progress to KS without treatment [1]. While commonly associated with alcohol use, WKS can occur with deficiencies in thiamine intake, absorption, storage, and metabolism from any etiology and has been associated with bariatric surgery, human immunodeficiency virus/acquired immune deficiency syndrome (HIV/AIDS), and hyperemesis gravidarum (HG).

HG presents a significant risk factor for WKS due to nutritional deficiency caused by persistent vomiting. Nausea and vomiting affect up to 80% of all pregnancies, and persistent vomiting is the most common indication for hospitalization during the first half of pregnancy. Symptoms become severe in up to 3% of these cases, resulting in weight loss, dehydration, and electrolyte imbalance [2, 3]. A recent systematic review of WE in pregnancy revealed a maternal mortality rate of 5% and 50% fetal mortality, some despite diagnosis and treatment [4]. In the general population, fetal demise after 20 weeks gestation occurs in about 1 in 175 pregnancies [5]. This increased mortality to the fetus is a unique and defining feature of HG-induced WKS.

While preventable, HG-associated WKS is considered rare and can be missed. We present the first-reported case of HG-associated WE in a patient in the first half of pregnancy in which a missed diagnosis led to septic shock, fetal demise, and eventual profound KS.

## Case report

**Fig. 1:**
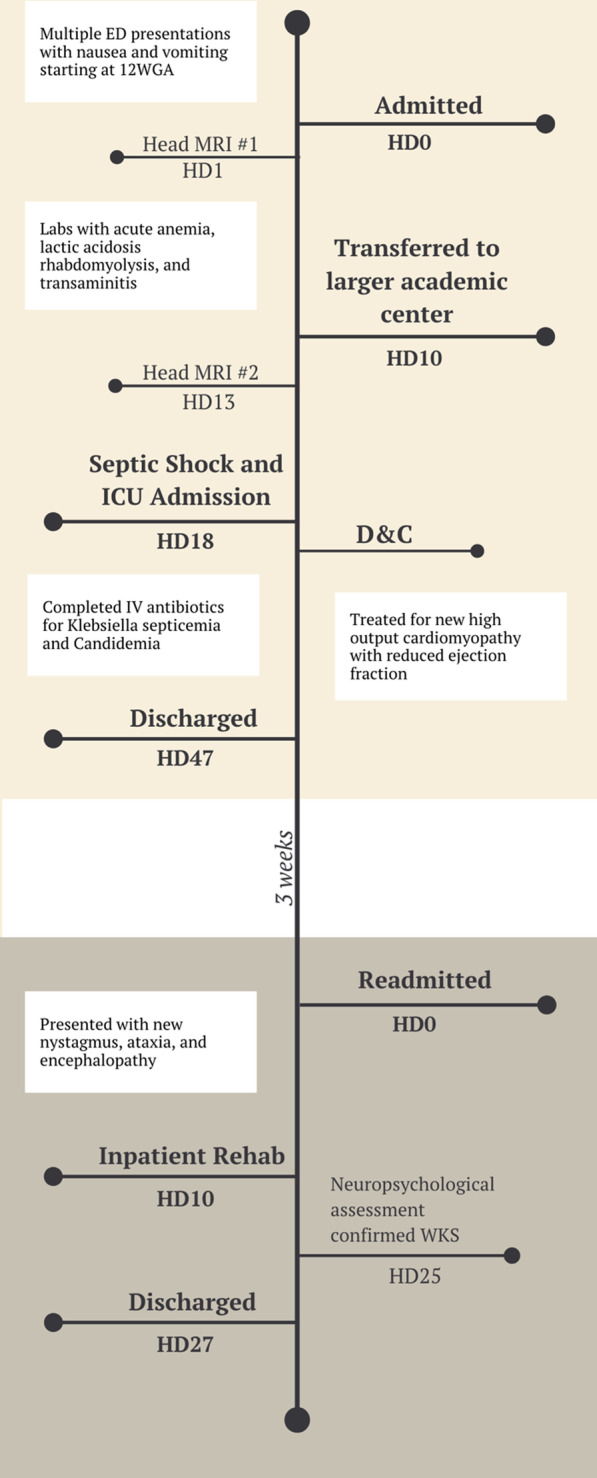
The patient was admitted at 15 weeks gestational age, progressing to sepsis and loss of the fetus by hospital day (HD) 18

A 33-year-old primigravid African American woman at 15 weeks gestation presented obtunded to the hospital for acute-onset altered mental status. Her medical history was significant for asthma, hypothyroidism, and β-thalassemia trait. She had no past psychiatric or substance use history. Her pregnancy had been complicated by ketonuria, nausea, vomiting, and poor oral intake noted at her initial prenatal visit with emergency department presentations for intractable vomiting and poor oral intake requiring intravenous fluid repletion (Fig. 1)*.*

On examination, the patient was disoriented, agitated, and persistently tachycardic to 150 beats per minute. Labs were notable for multiple electrolyte abnormalities, microcytic anemia, transaminitis, hypothyroidism, lactic acidosis, and rhabdomyolysis (Tables 1, 2, 3, 4, 5). Urine drug screen and blood alcohol level were negative. Urinalysis showed protein, ketones, and large amounts of bilirubin. Initial blood cultures showed one set positive for methicillin-sensitive *Staphylococcus aureus* (MSSA). Computerized tomography (CT) of the head was unremarkable. Electrocardiogram (EKG) showed sinus tachycardia with a heart rate of 151 beats per minute. Obstetric ultrasound confirmed a single, live, intrauterine pregnancy at appropriate gestational age. Altered mental status was attributed to dehydration and sepsis, and she was started on intravenous nafcillin, hypotonic fluids, and high-dose parenteral thiamine. A plasma thiamine level did not demonstrate deficiency (Table 6).

**Table 1:** Serial metabolic panels showed early electrolyte abnormalities in support of dehydration and malnutrition due to vomiting

	Normal ranges	HD0	HD2	HD10	HD18
Glucose	70–140 mg/dL	122		77	78
Sodium	129–148 mmol/L	147	145	139	140
Potassium	3.3–5.0 mmol/L	**2.8**	3.5	3.6	4.1
Chloride	97–109 mmol/L	**114**	**118**	109	**111**
Bicarbonate	18–26 mmol/L	23.0	20	20	18
BUN	3–13 mg/dL	7	3	7	6
Creatinine	0.4–0.8 mg/dL	0.7	0.5	0.4	0.5
Phosphate	2.5–4.6 mg/dL			3.6	3.0
Calcium	8.2–9.0 mg/dL	**10.4**	9.1	9.6	
Magnesium	1.5–2.2 mg/dL	**1.4**		1.6	2.0

Abnormal findings are in bold

Ketonuria also supported a picture of minimal oral intake

BUN, blood urea nitrogen; mmol, millimoles; mg, milligrams; dL, deciliter; L, liter; HD, hospital day

**Table 2:** The sudden drop in hemoglobin without known cause prompted a transfer to a larger academic hospital on HD10

	Normal ranges	HD0	HD2	HD10	HD18
WBC	5.6–14.8 K/µL	7.9	8.1	7.0	**34.4**
RBC	2.81–4.49 M/µL	4.77	3.37	3.53	3.52
Hemoglobin	9.7–14.8 g/dL	11.3	**8.2**	**8.5**	**9.2**
Hematocrit	30–39%	33.6	**23.6**	**27.7**	**28.7**
MCV	85.8–99.4 fL	70.5	70.2	79	82
RDW	12.3–14.7%	16.8	17	26	25.5
Platelets	155–409 K/µL	180	168	**326**	187

Abnormal findings are in bold

The patient developed septic shock on HD18 with associated leukocytosis, which was presumed due to endometrial, urinary, or intravenous line source

WBC, white blood cells; RBC, red blood cells; K, thousands; M millions; µL, microliter; fL, femtoliter; g grams; HD hospital

**Table 3:** Transaminitis in the setting of rhabdomyolysis (Table 4) confused the diagnostic picture and supported the need for transfer

	Normal ranges	HD0	HD2	HD10	HD18
Total bilirubin	0.1–0.8 mg/dL	0.7	0.4	0.3	0.6
ALT	2–33 U/L	**185**	**147**	**407**	**183**
AST	3–33 U/L	**90**	**85**	**194**	**79**
Alkaline phosphatase	25–126 U/L	57	50	61	81
Total protein	5.7–6.9 g/dL	**7.1**	5.6	6.6	**5.2**
Albumin	2.6–4.5 g/dL	3.2	**2.5**	3.4	**2.5**

Abnormal findings are in bold

Liver synthetic function remained intact, and imaging was unrevealing

AST, aspartate aminotransferase; ALT, alanine transaminase; mg, milligrams; g, grams; dL, deciliter; L, liter; U, units; HD, hospital day

**Table 4:** Early lactic acidosis and rhabdomyolysis further complicated the presentation

	Normal ranges	HD0	HD2	HD10	HD18
TSH	0.37–3.64 µIU/mL	**8.340**		11.58	4.53
FT3	4.0–4.2 pg/mL			167	
FT4	0.6–1 ng/dL	0.85		**1.4**	1.3
Ammonia	< 32 µmol/L	27			
Lactate	0.4–2.0 mmol/L	**2.5**		1.4	4.2
ESR	7–47 mm per hour	15		**99**	
CRP	0.4–20.3 mg/L	**6.6**		1.4 mg/dL (< 0.8 mg/dL)	
CPK	25–75 U/L	**1754**	**1993**	**157**	

Abnormal findings are in bold

TSH, thyroid stimulating hormone; FT3, free triiodothyronine; FT4, thyroxine; ESR, erythrocyte sedimentation rate; CRP, C-reactive protein; CPK, creatine phosphokinase; μIU, micro-international units; pg, picograms; ng, nanograms; mg, milligrams; μmol, micromoles; mmol, millimoles; mm, millimeter; dL, deciliter; L, liter; U, units; HD, hospital day

**Table 5:** There was concern for possible early presentation of hemolytic anemia given drop in hemoglobin

	Normal ranges	HD0	HD2	HD10	HD18
PTT	24.2–38.1 seconds	**23**		28	31
PT	9.5–13.4 seconds	**13.6**		13.3	**18.1**
INR	0.85–0.97	**1.19**			**1.59**
d-Dimer	320–1290 ng/mL			**1610**	**27,324**
Fibrinogen	291–538 mg/dL			**890**	**502**
LDH	80–447 IU/L			**347**	

Abnormal findings are in bold

Direct Coombs was negative, and peripheral smear was nonspecific. Hemolysis was later ruled out on HD10

PTT, partial thromboplastin time; PT, prothrombin time; INR, international normalized ratio; LDH, lactate dehydrogenase; ng, nanograms; mg, milligrams; mL, milliliter; dL, deciliter; L, liter; IU, international units; HD, hospital day

**Table 6:** Nutritional studies were unremarkable and misleading as plasma thiamine level was obtained following initiation of repletion

	Normal ranges	HD0	HD2	HD10	HD18
B12	130–656 pg/mL	**1568**		1708	
B9	2.7–17.0 ng/mL			13.6	
B1 (plasma)	8–30 nmol/L		**896**		
Iron	44–178 µg/dL		73	68	
TIBC	302–519 µg/dL		221.6	288	
Transferrin	220–441 mg/dL		**155**		
Ferritin	2–230 ng/mL		197.4		

Abnormal findings are in bold

Elevated free thiamine did confirm adherence to repletion while inpatient

**Fig. 2:**
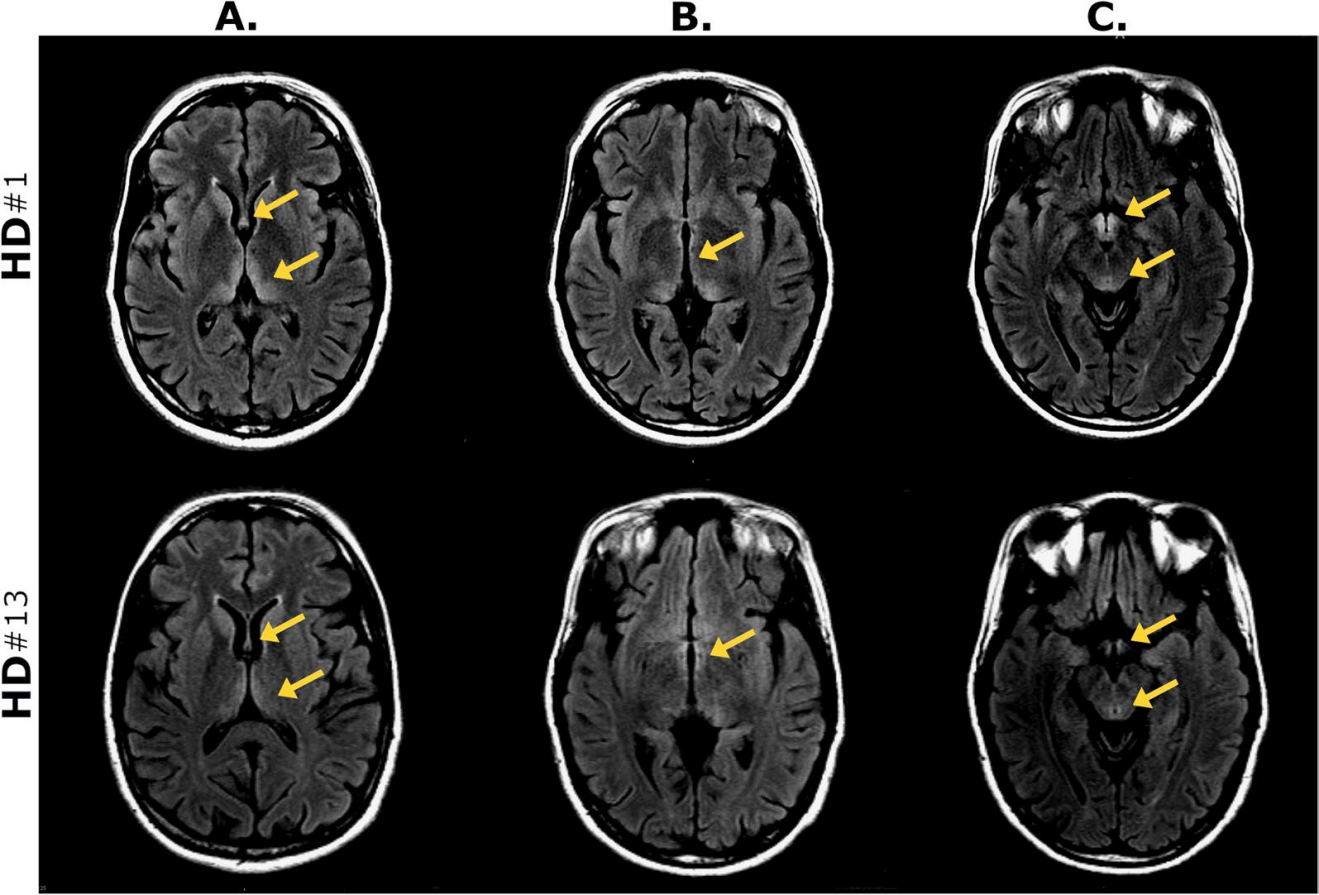
Magnetic resonance imaging (MRI) of the head using T2-weighted Fluid-Attenuated Inversion Recovery (T2/FLAIR) sequences obtained on hospital day 1 and repeated on transfer on HD13 demonstrates hyperintensities in the **A** fornix, bilateral medial thalami, **B** third ventricle, and **C** mammillary bodies and periaqueductal gray that leads to the floor of the fourth ventricle (not pictured). Aforementioned findings are demonstrated by the yellow arrows. The diagnosis of Wernicke Encephalopathy can be confirmed with these MRI scans demonstrating abnormalities; however, it is important to note that it is not sensitive as negative findings do not exclude the diagnosis of Wernicke–Korsakoff syndrome

**Fig. 3:**
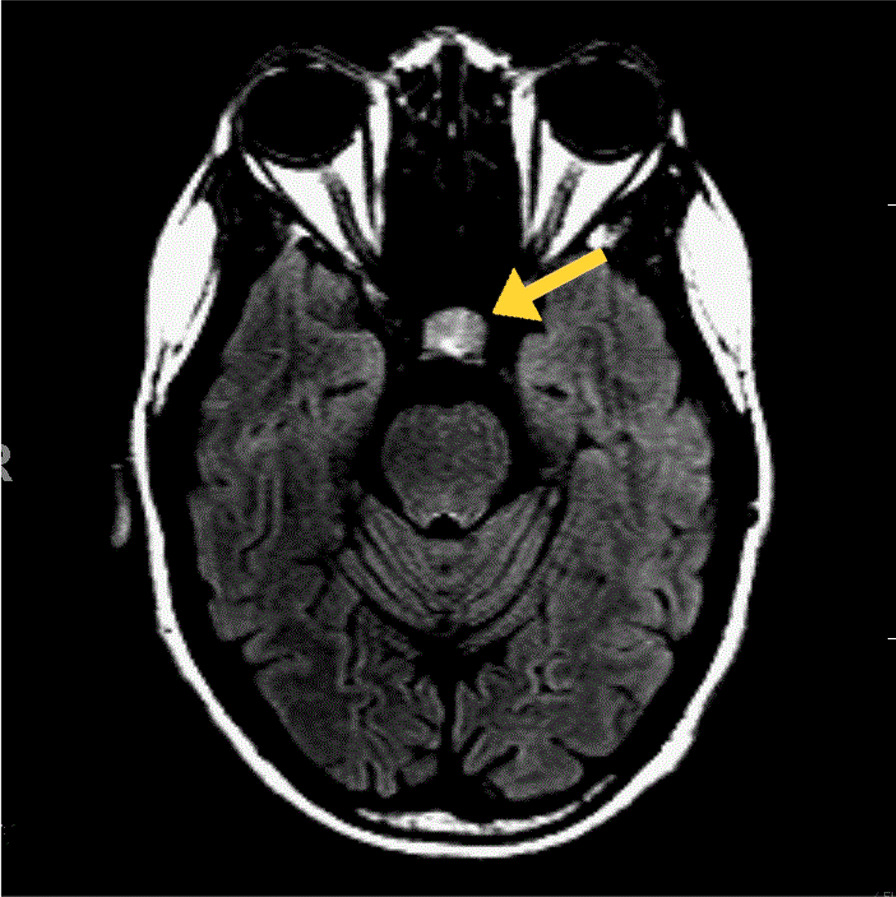
MRI head T2/FLAIR showing a hyperintense pituitary mass, demonstrated by the yellow arrow.

B12, Cobalamin; B9, folic acid; B1, thiamine; TIBC, total iron binding capacity; pg, picograms; ng, nanograms;

The patient developed paranoid delusions and auditory hallucinations prompting an encephalopathy workup. Rhabdomyolysis and transaminitis continued prompting concern for early hemolysis or rheumatological etiology (Tables 3 and 4). Sinus tachycardia persisted. Lumbar puncture showed elevated protein [glucose 58 mg/dL, protein 315 mg/dL, 2 white blood cells (WBC) per mm^3^, 32 red blood cells (RBC)  per mm^3^]. Viral hepatitis titers, paraneoplastic panel, copper level, and ceruloplasmin were unremarkable. Magnetic resonance imaging (MRI) of the head showed findings consistent with a diagnosis of WKS (Fig. 2). A new pituitary mass was noted (Fig. 3). Prolactin level was normal (234 ng/mL, normal range 110–330 ng/mL). Repeat blood cultures were negative.

She was transferred to a larger academic center on hospital day (HD) 10 for further evaluation. On examination, she was disoriented, confabulatory, paranoid, and surprised to hear she was pregnant. Transfer workup showed ongoing transaminitis, rhabdomyolysis, and hypothyroidism (Tables 3 and 4). Labs demonstrated new elevated fibrinogen and d-dimer (Table 5). Coagulopathy was ruled out by hematology consultants. Repeat blood cultures were negative. Transthoracic echocardiogram was unremarkable. She was seen by multiple consulting services, including infectious disease. MRI imaging of head, spine, and lower extremities failed to reveal a source as leukocytosis steadily worsened.

At HD18 and 18 weeks gestation, she developed septic shock with fetal demise and underwent dilatation and evacuation. She was intubated, started on pressors, and moved to the intensive care unit (Fig. 1). Blood cultures grew *Klebsiella* and *Candida*, and she was switched to piperacillin–tazobactam and anidulafungin. Repeat cardiac imaging showed new high-output cardiomyopathy with reduced ejection fraction of 15%. Heparin was infused due to concern for pulmonary embolism, which was ruled out by vascular medicine, and she was transitioned to enoxaparin. Transesophageal echocardiography did not demonstrate vegetation. Blood cultures cleared, and she was extubated and transitioned back to the general floors by HD28. On HD40 she spiked an isolated fever of 102 °F after completing intravenous antibiotics and her husband asked to take the patient home. In accordance with hospital risk management, the patient and family were counseled on the risks of her current medical condition and the patient was discharged with return precautions.

Three weeks later, the patient returned to the emergency department with her husband (Fig. 1). On physical examination, she demonstrated new horizontal nystagmus, ataxia, and encephalopathy. Urinalysis was notable for 3+ leukocyte esterase, positive nitrites, and > 182 WBCs per high-power field. Psychiatry consultants noted ongoing paranoia and cognitive impairment. Her husband as the surrogate decision maker agreed to continue treatment with ceftriaxone for a urinary tract infection and high-dose parenteral thiamine for presumed WKS. A central line was placed for long-term thiamine administration. The patient was transferred to acute physical rehabilitation and was incrementally able to participate in physical and occupational therapy. She received inpatient formal neuropsychological testing where she demonstrated profound anterograde amnesia, abulia, disorientation, impaired processing speed, and anosognosia highly consistent with a diagnosis of WKS. After 6 weeks of parenteral thiamine since her initial presentation until treatment plateau and 18 days of physical rehab, she was discharged back home with her husband.

## Discussion

Although WKS is classically found in patients with chronic alcohol use, this syndrome may be found in any patient with depleted intracellular thiamine such as from prolonged parenteral nutrition, gastrointestinal carcinoma, HIV/AIDS, eating disorders, chronic diarrhea, or persistent vomiting [6]. WKS is a clinical diagnosis, and identification depends on the clinical suspicion of those who may be looking for the classic constellation of ophthalmoplegia, confusion, and gait ataxia [7]. While these criteria were originally conceived to prevent underdiagnosis of WKS in alcoholics in which the syndrome frequently overlapped with hepatic encephalopathy, it continues to be utilized for diagnosis of WKS in a variety of patient populations [8]. Autopsy studies reveal, however, that up to 80% of cases of WKS are undiagnosed using these criteria; nearly 20% of patients present with none of these classical signs, and up to 37% may present with only one [9–11].

There are no reliable and accessible measures for thiamine deficiency despite biochemical changes often preceding overt physical signs. Tests of nutritional status including 24-h urine excretion of thiamine, and specialty blood tests have been developed but are of limited clinical use given necessary processing time. Free thiamine in plasma or serum is representative of recent intake rather than storage and can be useful in assessing adherence. If available, thiamine in whole blood is less sensitive to recent intake and will provide a more accurate assessment of thiamine deficiency. MRI findings are only 53% sensitive but are 93% specific [12]. In this case, the patient’s free thiamine was appropriately elevated following initiation of replenishment and the lack of supporting evidence for WKS further confused the initial diagnostic impression. After transfer to a larger institution, diagnosis was confirmed with MRI and neuropsychological assessment. WKS remains a clinical diagnosis and requires the synthesis of history, physical examination, and objective findings.

HG is a preventable risk factor for nonalcoholic WKS. Although it generally takes months to years for an alcoholic to develop overt signs of WKS, the same syndrome in a pregnant woman may present as early as 6 weeks gestation and progress rapidly due to increased thiamine requirement during pregnancy [4]. Thiamine requirement increases by as much as 45% for fetal growth and development [13]. Measures of free thiamine have been shown to be higher in cord blood than in maternal blood, suggesting that the fetus is able to sequester thiamine at the expense of the mother [14]. While there is no direct evidence that thiamine deficiency of the mother can cause fetal complications, WKS is associated with fetal demise and spontaneous abortion [15].

Diagnosis of WKS should not rely upon the presence of the classical triad without consideration of the pregnancy and nutritional status of the patient. The patient did not exhibit oculomotor or cerebellar abnormalities early in her presentation. She presented at 12 weeks gestational age with HG, which is inconsistent with the typical prolonged nutritional deficiency seen in alcoholics. She went on to demonstrate several characteristics consistent with a diagnosis of WKS including profound anterograde amnesia, abulia, disorientation, impaired processing speed, and anosognosia along with peripheral neuropathy and high-output cardiomyopathy, also known as beriberi [16, 17]. It is unclear if the fetal demise was directly caused by her thiamine deficiency, though the resultant sepsis greatly accelerated her course as metabolic demand increased. Additional evidence supports that severe infection may be common in thiamine deficiency and repletion may be therapeutic [18–21].

The outcome may have been different if thiamine supplementation was initiated as soon as nutritional deficiency was suspected. In total, there were 3 weeks from initial presentation at 12 weeks gestational age until the patient arrived obtunded at 15 weeks gestational age and was admitted. There are no standardized national guidelines for thiamine administration, though the European Federation of Neurological Societies and Royal College of Physician both advocate for thiamine 500 mg intravenously, three times a day until acute WE has resolved and benefits plateau, which may take several months [22]. These guidelines may need to be adapted in pregnancy, with earlier and more aggressive treatment.

## Conclusions

This patient’s initial presentation was medically complex and severe, and presented numerous diagnostic challenges. The multisystem complications seen in severe thiamine deficiency can delay timely administration of high-dose thiamine, particularly in pregnancy, in which the classic triad of symptoms may not be sensitive enough due to the rapid progression of neuropsychiatric symptoms in this population. We advise a low threshold for high-dose parenteral thiamine repletion in pregnant women with persistent vomiting as HG-induced severe thiamine deficiency may result in WKS, sepsis, and fetal demise.

## Data Availability

Not applicable.

## Abbreviations

WE: Wernicke encephalopathy
KS: Korsakoff syndrome
WKS: Wernicke–Korsakoff syndrome
HD: Hospital day
